# Investigation on the potential of applying bio-based edible coatings for horticultural products exemplified with cucumbers

**DOI:** 10.1016/j.crfs.2022.100407

**Published:** 2022-12-05

**Authors:** G. Rux, C. Labude, W.B. Herppich, M. Geyer

**Affiliations:** a Department of Horticultural Engineering, Leibniz Institute for Agricultural Engineering and Bioeconomy (ATB), Max-Eyth-Allee 100, 14469, Potsdam, Germany; b Department Life Sciences and Technology, Berliner Hochschule für Technik (BHT), Luxemburger Straße 10, 13353, Berlin, Germany

**Keywords:** Shelf life, Perishables, Transpiration, Mass loss, Consumer survey

## Abstract

Plastic packaging for fresh horticultural produce has many advantages but generates plastic waste and ecological alternatives are required. Edible coatings can retard many processes related to loss of quality. Hydrophobic lipid-based coatings are preferably applied for fresh fruits and vegetables. The approval of such coatings for products with edible peels in EU is increasingly under discussion. However, investigations on the efficiency of various edible coatings on soft-skinned fruit and vegetables are rare and it is currently unclear whether the consumer will accept them. Therefore, this study investigates (1) important characteristics of a lipid-based coating and (2) its ability to maintain the post-harvest quality of fresh cucumbers. This was evaluated by a comparative storage test under common suboptimal retail conditions (20 °C; 65% RH). The study also evaluates (3) the general perception of consumers about and their acceptance of the application of edible coatings on fresh fruit and vegetables with edible peels. The investigated coating was able to drastically reduce water loss (54–68%) and fruit respiration (approx. 33%) of fresh cucumber. The reduction of tissue stiffness was delayed by 2 days, thus, prolonged shelf life. Majority of consumer (77%) endorse the application of edible coatings as an alternative to plastic packaging, but emphasized important requirements for them.

## Highlights

• Coatings applied provide effective barriers for O_2_, CO_2_ and water vapor transfer.
• Coating application on cucumbers drastically reduced water losses.
• Application of coatings did not negatively affect quality of cucumber.
• a consumer survey indicated preferences for edible coatings over plastic packaging.
• washability and low perceptibility were identified as important requirements.

## Graphical abstract

**Figure:**
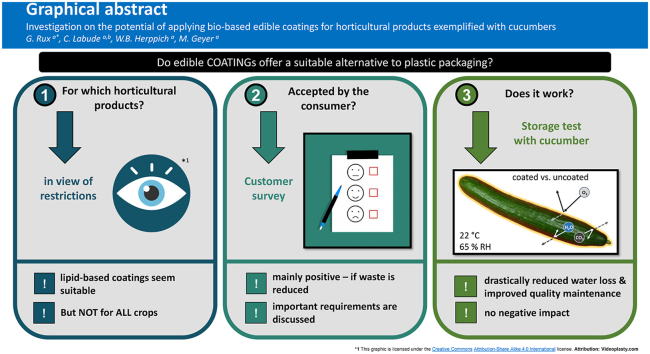

## 1 Introduction

Extensive conventional packaging of fruit and vegetables generates huge amounts of plastic waste, which results in serious ecological problems. In recent years, social pressure to reduce the application of plastic materials largely increases. This motivates industry to looking for suitable alternatives. Hence, edible coatings have been subject of scientific investigations since many years, besides biodegradable or compostable plastics, paper or cardboard (e.g. Mohamed et al., 2020; Blancas-Benitez et al., 2022). Indeed, it was shown that optimized edible coatings may avoid packaging waste but maintain quality and improves shelf life of fresh produce (White and Lockyer, 2020; Armghan Khalid, 2022). Coating membranes on the product surface mainly lower the exchange of water vapor, O_2_, CO_2_ or ethylene by forming a barrier against gas diffusion (Li and Barth, 1998; Dhall, 2013). In fresh fruit and vegetables, this reduced diffusion may control and modify the internal concentrations of these gases, eventually retarding physiological processes related to quality loss and product degradation in postharvest. Edible coatings, therefore, present an alternative to modified atmosphere storage (Dhall, 2013). Many studies investigated the various effects of coatings on product physiology. These include the reduction of water losses (Avena-Bustillos et al., 1997), respiration, ethylene production, and the connected metabolic degradation of value-added compounds (Thompson 2003; Lin and Zhao, 2007). Coatings may also lower loss of tissue firmness (Lin and Zhao, 2007), and retard solute movement (Li and Barth, 1998), enzymatic oxidation, browning discoloration (Park, 1999; Lin and Zhao, 2007), and microbial decay. In addition, coatings may protect from physical damages (Lin and Zhao, 2007).

Lipid-based coatings are highly hydrophobic and, thus, form excellent barriers against water (vapor) diffusion (Dhall, 2013; Hassan et al., 2018). Therefore, they are applied favorably on fresh fruits and vegetables particularly susceptible to water losses. In this context, coatings often synergize with natural surface waxes. These lipid-based coatings can be formed from neutral lipids including acetylated monoglycerides and fatty acids, natural waxes and resins (Morillon et al., 2002; Min and Krochta, 2005; Lin and Zhao, 2007). Many of these compounds can be produced from environmentally friendly plant materials (Poverenov et al., 2018).

Except apple waxing, current EU regulations permit the application of edible coatings only for horticultural products with inedible peels. Actually, lipid-based coatings (mono- and diglycerides, E471) are only approved for citrus fruits, pomegranate, melon, pineapple, banana, papaya, mango and avocado. However, many other countries already classified several edible coatings as generally consumer safe. Food retailers are therefore in favor of changing the legal situation, which meanwhile appears not seem unlikely. In this context, consumer acceptance is a necessary prerequisite for future use of edible coatings (Wan et al., 2007; Yousuf et al., 2021). Without the acceptance by a majority of consumers, a market launch seems inconceivable. Currently, it is unclear whether and in what form the consumer will accept edible coatings on fresh horticultural products. Hence, comprehensive knowledge of relevant consumer expectations and requirements plays a decisive role for future developments in this field.

Based on relevant lacks of knowledge, the present study investigates **(1)** the physical properties of a currently available lipid-based edible coating. This includes the detailed analysis of its efficacy as barrier against water vapor, O_2_ and CO_2_ diffusion. In addition, **(2)** the ability of this coating to maintain the post-harvest quality of cucumbers was evaluated by performing a comparative test with coated and uncoated cucumbers stored under common suboptimal retail conditions (20 °C; 65% RH) for up to eight days. Cucumbers where selected because transpiration is the major cause of quality losses. Furthermore, due to the important relevance as necessary prerequisite for future use, **(3)** the general perception of consumers about edible coatings on fresh fruit and vegetables and their potential marketability was assessed in detail by a customer survey.

## 2 Material and methods

### 2.1 Characterization of lipid-based coating

#### 2.1.1 Coating material

For this study, the commercially available lipid-based coating LiquidSeal (Liquidseal B.V., Leiden, Netherlands) was used in different formulations. The manufacturer recommends this coating for avocado, mango, citrus fruits and other products with a hard non-edible skin. Main components of this coating are neutral glycerides, i.e., esters of glycerol and fatty acids, primarily with carbon chain length of 16 to 18 C-atoms.

#### 2.1.2 Water vapor permeability

**Fig. 1:**
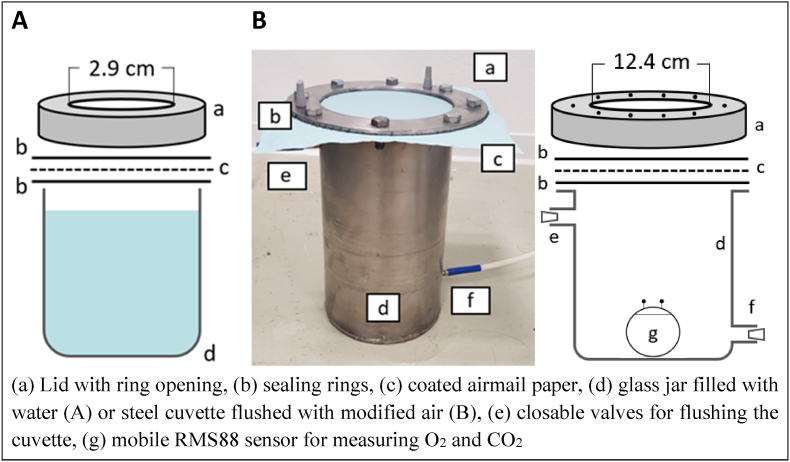
Schema of the system applied for transmission measurement for water vapor (**A**) and O_2_/CO_2_ (**B**).

Water vapor transmission rate (TR_WV_) and permeability (P_WV_) of the lipid-based coating were determined modified after ASTM (2012) Standard Test Method E96/E96M. Accordingly, the mass loss of a water-filled jar through a coated barrier layer was measured gravimetrically. For this, round openings (diameter 2.9 cm) were cut into the lid of standard 100 mL laboratory screw-in jars (Rasotherm® GL 45; DWK Life Sciences, Wertheim, Germany). Airmail paper (37 g m^−2^; MK 54 260; Brunnen Papier GmbH, Stuttgart, Germany) was coated by spraying with LiquidSeal and placed on the opening between two sealing rings (inner/outer diameter = 29/40 mm). The jar was filled with 50 ml distilled water and tightly closed with the lid (Fig. 1A). The total exchange area was 6.6 cm^2^. Ten jars were placed in a climat chamber (IPP260plus, Memmert GmbH & Co KG, Schwabach, Germany), with ambient humidity and temperature held constant at 30% rH and 35 °C, respectively. The mass loss of each jar was measured 3-times over a total time of 8 d. Transmission rate and permeability of the coating were calculated as:

(1) TR_WV_ (mg cm^−2^ h^−1^) = Δm / (A ∙ Δt)

with Δm = mass loss (mg), A = exchange area (cm^2^) and Δt = duration of measurement (h), and

(2) P_WV_ (mg μm kPa^−1^ cm^−2^ h^−1^) = TR_WV_ ∙ Δx / ΔP_H2O_

with Δx = thickness of coating (μm) and ΔP_H2O_ = water vapor partial pressure difference (kPa).

#### 2.1.3 O_2_ and CO_2_ permeability

O_2_ and CO_2_ transmission rate (TR_O2_; TR_CO2_) and permeability (P_O2_; P_CO2_) were determined with a similar method. A 2.9 L steel cuvette was covered with spray-coated airmail paper giving the total exchange area of 122.8 cm^2^ (Fig. 1B). Six cuvettes were flushed with modified air (0 kPa O_2_ + 20 kPa CO_2_) and decrease or increase of internal O_2_ and CO_2_ partial pressure due to gas transmission through the coated paper were monitored with mobile RMS88-sensors (Keshri et al., 2019) placed inside each cuvette. All cuvettes were kept at 20 °C and normal air concentrations (20.9 kPa O_2_ + nearly 0 kPa CO_2_). The transmitted O_2_ and CO_2_ volumes were calculated between the range of O_2_ partial pressure of 2–17 kPa and the corresponding CO_2_ partial pressure for all measurements as:

(3) ΔV_O2/CO2_ = ΔO_2_/CO_2_ ∙ V_cuvette_

with ΔV_O2/CO2_ = total exchange of O_2_ and CO_2_ (mL), ΔO_2_/CO_2_ = difference of O_2_ and CO_2_ partial pressure and V_cuvette_ = volume of the cuvette (mL). TR_O2/CO2_ and P_O2/CO2_ of the coatings were calculated as:

(4) TR_O2/CO2_ (mL cm^−2^ h^−1^) = ΔV_O2/CO2_ / (A ∙ Δt)

with ΔV = total exchange of O_2_ and CO_2_ (mL), A = exchange area (cm^2^) and Δt = duration of measurement (h), and

(5) P_O2/CO2_ (mL μm kPa^−1^ cm^−2^ h^−1^) = TR_O2/CO2_ ∙ Δx / ΔP_H2O_

with Δx = thickness of coating (μm) and ΔP_O2/CO2_ = partial pressure difference of O_2_ and CO_2_ (kPa).

In addition, the mass of dried coating on the spray-coated airmail paper was determined gravimetrically and the film thickness calculated based on the density (920 kg m^−^³).

### 2.2 Storage test with cucumber

#### 2.2.1 Material

For storage tests, fresh cucumbers of a common, local variety (*Cucumis sativus* L. ‘Prolog’) were obtained from a local grower and transported to the Department of Horticultural Engineering, Leibniz Institute for Agricultural Engineering and Bioeconomy, Potsdam, Germany (ATB). Masses and lengths of cucumbers ranged between 410g and 550 g, and 251 mm and 327 mm, respectively, with means of 457.5 ± 31.9 g and 287 ± 20 mm. Before each experiment, samples could equilibrate to a fruit temperature of 20 °C within 2 h.

#### 2.2.2 Sample preparation, storage and sampling

Cucumbers were randomly divided into two batches of 15 fruit each and either treated with water (control) or Liquid Seal (coating) by immersion. Then fruit were dried on a mesh (1 cm × 1 cm mesh width) under free convection at 20 °C and 65% RH for approx. 1 h. Afterwards, all cucumbers were stored on similar meshes for up to eight days at 20 °C and 65% RH to simulate retail display conditions. Mass loss, CO_2_ release, modulus of elasticity and potential maximum photochemical efficiency (F_v_/F_m_) of all fruit were non-destructively determined at the beginning and on storage days 2, 4 and 8.

#### 2.2.3 Determination of mass loss

The mass of each cucumber was determined daily with an electronic balance (CPA1003S, Sartorius AG, Göttingen, Germany) and relative mass losses (%) calculated based on the initial mass.

#### 2.2.4 Modulus of elasticity

The modulus of elasticity (E) was obtained non-destructively by quasi-static compression test using a SMS XT Plus texture analyzer (Stable Micro Systems, Godalming, UK) fitted with a spherical steel body (12.7 mm diameter). Tissue deformation was recorded at a fixed compression force (F) of 1 N (test speed 3 mm min^−1^; trigger force 0.1 N). Each cucumber was measured in triplicate around its center equator and E calculated according to ASAE (1999) as

(6) $$E=0.531\times F\times \left(1-\mu \right){\times \left(\frac{2}{R}+\frac{4}{d}\right)}^{0.5}$$

with μ = poisons ratio = 0.49 (Mohsenin, 1986), d = diameter steel body of 12.7 mm and R = radius of the cucumber at the measuring position.

#### 2.2.5 Measurement of CO_2_ release

The CO_2_ release was measured with a custom-made gas exchange system (for details see Rux et al., 2017). Briefly, two cucumbers were placed in an acrylic glass cuvette (8.2 L), and the increase of the CO_2_ partial pressure within the cuvette measured with a GMP222 CO_2_ sensor (Vasalia, Helsinki, Finland) and recorded with a NetDAQ 2645A data logger (Fluke Deutschland GmbH, Glottertal, Germany). In total eight cucumbers of each treatment (n = 4 per treatment) were analyzed at 20 °C on each sampling day. CO_2_ release rates (mg kg^−1^ h^−1^) were calculated based on the mass increase of CO_2_ (mg) and related to the product mass (kg), and the duration (h) of measurement (Caleb et al., 2016).

#### 2.2.6 Chlorophyll fluorescence

The potential maximum photochemical efficiency of photosystem II (Matyssek and Herppich, 2018) was evaluated with an open PSI FluorCam FC 800-O chlorophyll fluorescence imaging system (Photon Systems Instruments, Drasov, Czech Republic) following Herppich et al. (2020). From the basic (F_0_) and the maximum (F_m_) fluorescence signals of dark-adapted (approx. 15 min) cucumbers, the variable fluorescence (F_v_ = F_m_ − F_0_) was calculated pixel-wise and the potential maximum photochemical efficiency computed as F_v_/F_m_.

### 2.3 Customer survey

Data were collected both online and in retail store. There were no restrictions to participate in the survey. The survey was therefore conducted according to the stochastic (random) selection method, where the sample generally, with maximum accuracy, reflects the structure of the entire population. The representativeness of the data depended on the absolute sample size, as the stochastic error can be attributed to the entire whole population. The information relevant to the evaluation was collected by using a questionnaire, including open and closed questions, but also general information on edible films and coatings.

In total 148 adults between “under 20” and ”over 65 years” participated in the survey, with only one incomplete set of answers being submitted. Apart from a minimum age of 16 years, there were no exclusion criteria for participation in the survey. The largest proportions of participants were between 20 and 30 years (44%), and 30 and 45 years (29%) old. Women made up the majority of respondents (62%). The size of households in which participants lived varied pronouncedly, however a predominance of single (27%) and 2-persons (35%) households was obvious.

### 2.4 Statistical analysis

To determine the water vapor permeability of used lipid-based coating, 10 replicates were measured over a total of 3 d (n = 10). O_2_ and CO_2_ permeability was determined with six repetitions (n = 10). In the storage test with cucumber, for each variant (control and lipid-based coating) and sampling day, 15 replicates (n = 15) were analyzed for each quality parameter, with exception of CO_2_ release. Here, a single measured value was determined as the mean of two samples. A total of 4 single measured values (n = 4) were determined for each variant and sampling day.

Statistical analyses (ANOVA) were performed with WinSTAT (R. Fitch Software, Staufen, Germany) and all results presented as means ± standard deviation (SD). Duncan's multiple range test (α = 0.05; p < 0.05) was used to determine the significance of the differences between means.

## 3 Results and discussion

### 3.1 Characterization of lipid-based coating

The lipid-based coating used in this study showed acceptable adhesive properties and fairly uniform distribution on the treated surfaces. The means of the masses of dried coatings on a treated surface was 2.0 ± 0.31 mg cm^−2^, corresponding to a film thickness of about 22.2 ± 3.3 μm. After application, the coating dried within a moderate time of less than 1 h at 20 °C and 65–70% RH under free convection.

Compared to uncoated airmail paper, coating reduced the permeability of all investigated gases significantly. The average permeability of the tested lipid coating samples was 17 ± 1.68 mg μm kPa^−1^ cm^−2^ h^−1^ for water vapor, 1.49 ± 0.36 ml μm kPa^−1^ cm ^2^ h ^1^ for O_2_ and 1.09 ± 0.26 ml μm kPa^−1^ cm ^2^ h ^1^ for CO_2_ (Table 1). Thus, the lipid-based coating formed an effective diffusion barrier, in contrast to polysaccharide- and protein-based coatings (Greener and Fennema, 1992). The water vapor permeability of the present coating is similar to that of commercial wax coatings, which range between 0.5 and 45.9 mg μm kPa^−1^ cm^−2^ h^−1^ (Hagenmaier and Shaw, 1992).

**Table 1:** Water vapor and O_2_/CO_2_ transmission through airmail paper with and without coating.

Parameter	TR (mg/mL cm^−2^ h^−1^)	P (mg/mL μm kPa^−1^ cm^−2^ h^−1^)
H_2_O (mg)	3.01 ± 0.30	16.51 ± 1.65
O_2_ (mL)	1.85 ± 0.44	4.24 ± 0.96
CO_2_ (mL)	1.43 ± 0.35	3.12 ± 0.70

In the present study, the measured permeability of both O_2_ (0.15–13.00 ml μm kPa^−1^ cm^−2^ h^−1^) and CO_2_ (0.55–105 ml μm kPa^−1^ cm^−2^ h^−1^) were in the lower ranges of commercial wax coatings (Hagenmaier and Shaw, 1992). As a result, the coating is potentially able to modify the atmosphere inside fruit resulting in relatively high CO_2_ and low O_2_ concentrations, which possibly retards ripening and extends shelf life (Banks et al., 1993; Lin and Zhao, 2007). However, the low permeability of these gases could also induce the undesirable switch to anaerobic metabolism, as observed with coatings of very low permeability (Yearsley et al., 1996). This was, indeed, recorded with wax and shellac coatings, which resulted in the formation of high concentrations of ethanol and acetaldehyde (Petracek et al., 1998; Alleyne and Hagenmaier, 2000).

Generally, gas exchange between the fruit and the surrounding atmosphere occurs both via cuticle and various epidermal pores (Banks et al., 1993). It has been pointed out, however, that coatings may mainly block the pores (Banks et al., 1993). Consequently, the total gas exchange and the resulting internal atmosphere of the product is not only dependent on the coating permeability. Rather, it results from the interaction of both, the surface structure of the product (number and size of pores) and the properties of the coating (viscosity, surface tension and other factors) (Banks et al., 1993). Therefore, the exclusive measurement of the coating permeability only allows a rough prediction of the resulting internal gas composition. Horticultural products vary greatly in terms of surface structure and properties, thus, the suitability of a lipid-based coating must be evaluated individually for each horticultural product.

### 3.2 Storage test with cucumber

**Fig. 2:**
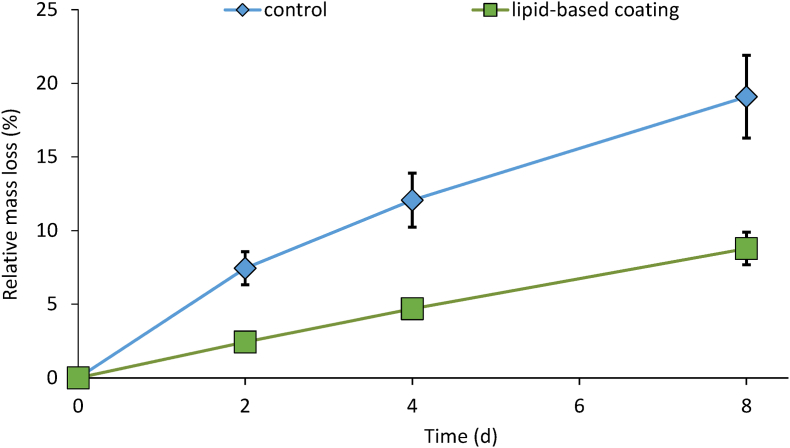
Relative mass losses of coated and uncoated cucumbers stored under simulated retail conditions at 20 °C and 65% RH for up to 8 d.

During storage, both uncoated and coated cucumbers continuously but decreasingly lost mass, though this effect was more distinct in the controls (Fig. 2). These mass losses were mostly due to transpiration, as this constitute approx. 90% of total mass loss (Ben-Yehoshua, 1987). Mean relative mass loss of controls was 7.4% already after two days and increased to 19.1% after 8 d of storage. The lipid-based coating drastically, by 54–68%, reduced relative water losses to approx. 32% that of controls on the second day, and to 46% on day eight of storage.

The effective reduction in mass loss due to the application of the coatings may also be explained by the storage temperature of 20 °C, because, generally, storage temperatures could affect the efficiency of the coatings. In carnauba wax-coated cucumbers, the coating was rough and covered with wax crystals at 10 °C, while it shows a smooth and homogeneous surface at 25 °C (Gutiérrez-Pacheco et al., 2020). Crystallization at low temperatures made the wax more brittle and permeable to water vapor (Bosquez-Molina et al., 2003; Endlein and Peleikis, 2011; Doan et al., 2018). Thus, Gutiérrez-Pacheco et al. (2020) suggested to store wax-coated fruit at higher temperatures to avoid crystallization, as also has been proposed Satsuma mandarins (Won and Min, 2018), Valencia oranges (Motamedi et al., 2018) and papaya fruit (Miranda et al., 2019).

**Fig. 3:**
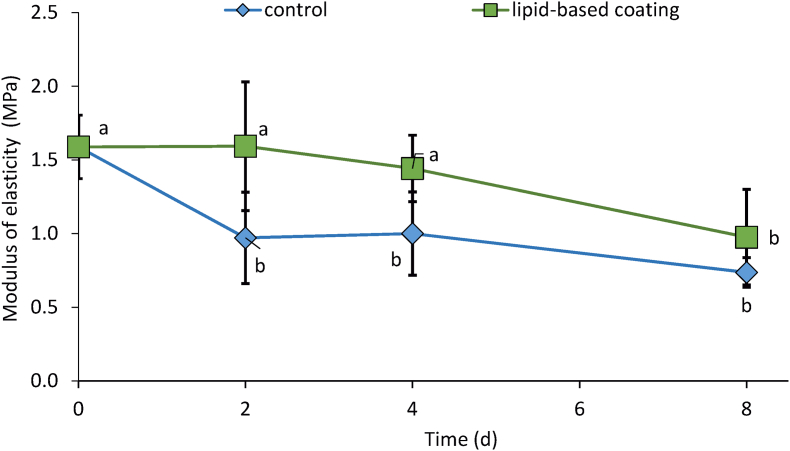
Modulus of elasticity of coated and uncoated cucumbers stored under simulated retail conditions at 20 °C and 65% RH for up to 8 d.

Also, storage had a significant effect on the elastic modulus (E, Fig. 3). In controls, E declined by approx. 40% within only two days of storage and by 50% after 8 d compared to the initial value. In coated cucumbers, however, E significantly declined to 40% only on day 8. Similarly, in cucumbers stored at 25 °C and 35% RH for up to 9 d (Jahangiri et al., 2016), the elastic modulus decreased continuously to 39%. However, the authors did not determine mass losses, which prevented a detailed comparison of effects.

The modulus of elasticity, which characterizes the stiffness of a product (Mohsenin, 1986; ASAE, 1999), is useful in non-destructively describing changes in texture and, thus, product quality. Short-term reduction of elastic modulus or stiffness is primarily due to water loss-induce cell or tissue turgor decline (Herppich et al., 1999), while during long-term storage, it is also associated with changes in the chemical and biophysical properties of cell walls (Zhou et al., 1999; Herppich et al., 2000). Thus, particularly during short-term storage, humidity and temperature drastically may influence texture changes (De Smedt et al., 2002; Herppich et al., 2003; Hertog et al., 2004).

**Fig. 4:**
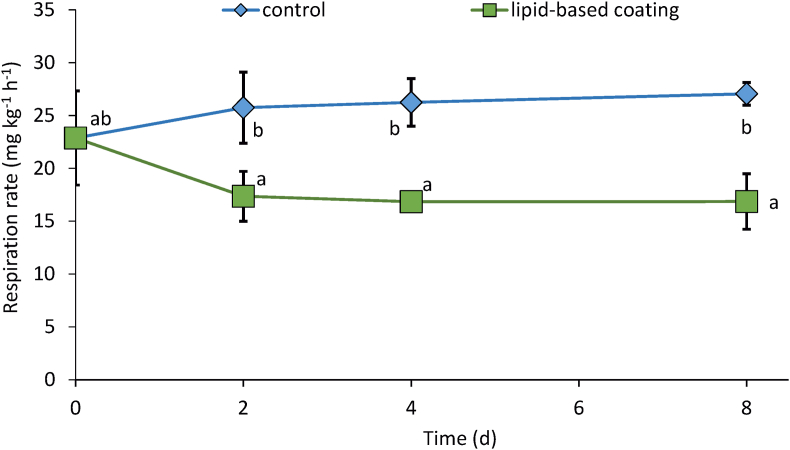
CO_2_ release rates of coated and uncoated cucumbers stored under simulated retail conditions at 20 °C and 65% RH for up to 8 d.

Respiration rates of controls slightly and insignificantly increased during storage (Fig. 4). The CO_2_ release of coated cucumbers, on the other hand, insignificantly declined compared to the initial rates (22.9 ± 4.5 mg kg^−1^ h^−1^). Both minor effects, however, resulted in significantly lower rates of CO_2_ release in coated cucumbers, potentially indicating a lower respiration in these samples. Reduced respiration can be associated with lower metabolic activity, e.g., senescence, and/or probably result from the reduced O_2_ availability (Kader and Saltveit, 2003; 2010). The lower CO_2_ release rates may also be related to the coating-induced lower CO_2_ permeability, which resulted in increased CO_2_ concentrations within the fruit. These changes of internal gas concentrations, i.e., increased CO_2_ and decrease O_2_ concentration, finally reduce the respiration activity, which is known to prolong shelf life (Saltveit, 2019). Anyway, there are no signs that the coating tested in this study disturbed aerobic respiration but has a positive effect on the overall quality of stored cucumbers.

This agrees well with the results of the chlorophyll fluorescence analyses. The initial value (0.74 ± 0.03) of potential maximum photochemical efficiency, F_v_/F_m_, remained unaffected for both coated (0.74 ± 0.05) and uncoated (0.74 ± 0.02) cucumbers throughout the storage. This indicates the intactness of the photosynthetic machinery (Matyssek and Herppich, 2018), which reflects a high internal quality of the fruit.

### 3.3 Customer survey

The aim of the survey was to assess generally the consumer perceptions toward plastic packaging and, in particular, toward the acceptability of edible coatings on horticultural products with edible peel. The survey showed a broad distribution in terms of age, gender and household size. The overrepresentation of women was in line with the fact that women are still often mainly responsible for shopping and, therefore, represent well the average customer. The majority of respondents (73%) belonged to the age groups below 45 years. Although this does not represent the real distribution in the population, it is nevertheless of particular importance for assessing customer opinion. These age groups form the future majority of the population and demands from this group will become increasingly relevant. The high proportion of single (27%) and two-person households (35%) is typical for urban areas in Germany. However, the number of single households tended to increase during recent decades (Statistisches Bundesamt., 2021). Nevertheless, it should be noted that, as in all surveys, there was an overrepresentation of basically open and interested persons among the survey participants who could be motivated to participate at all. It can be assumed that the respondents had an above-average knowledge of nutrition, health and the environment, which was reflected by the fact that when buying fruit and vegetables, they primarily buy organic, seasonal and/or regional products (Q1, Table 2).

**Table 2:** Results of the consumer survey; questions with given answer options (n = 148).

Question		Proportion
**What do you primarily look for when buying fruits & vegetables?**		
Q1	*I buy organic products*	47.3%
	*I buy seasonal and/or regional products*	70.3%
	*I pay attention to the quality of the products*	80.4%
	*I pay attention to a low price*	33.8%
	*None of the above criteria is decisive for my purchase*	2.0%
**How would an edible protective film on fruits and vegetables influence your purchase decision?**		
**Q2**	*I would prefer to buy such products*	59.5%
	*Such a protective film would not play a role in my purchase decision*	18.2%
	*I would prefer products without such a protective film*	16.2%
	*I would not buy such products*	2.0%
	*I do not know*	8.8%
**In my opinion, an edible protective film on fruits and vegetables should …**		
**Q3**	*be completely washable*	68.2%
	*not be perceptible on the product*	48.0%
	*be shown in detail on the label*	50.7%
	*lead to lower prices of fruits & vegetables*	16.9%

Overall, the majority of respondents was strongly to very strongly (78.8%) bothered by the packaging waste generated (Q4, Table 3). This once again highlights the relevance described at the beginning for the development of suitable alternatives. The basic idea of covering fruits and vegetables with an edible protective film to avoid plastic packaging was viewed positively by the majority of respondents (77%; Q5, Table 3). Furthermore, 59.5% of respondents would prefer to buy products with a coating and only 2% would not want to buy such products in principle (Q2, Table 2). However, it also became clear from the questionnaire that the preference for fruits and vegetables with coating essentially related to products packaged in plastic, i.e., as an alternative to packaging. Most respondents indicated a preference for completely unpackaged products over products with coating. A clear prioritization in the order “unpackaged” > “with coating” > “plastic packaging” was often communicated. Accordingly, the use of coatings appeared to be relevant above all for products that are generally only offered packed. The use of coatings for regional products with short transport distances, on the other hand, was viewed critically.

**Table 3:** Results of the consumer survey; questions with rating scale (n = 148).

Question		1	2	3	4	5	6	7
**Q4**	**Does the packaging waste generated when buying fruit & vegetables bother you?**	1.4%	0.7%	1.4%	2.7%	6.1%	22.3%	65.5%
	(1 = not at all; 7 = yes a lot)							
**Q5**	**What do you think about covering fruits and vegetables with an edible film?**	5.4%	4.7%	6.1%	6.8%	16.9%	23.6%	36.5%
	(1 = not good; 7 = very good)							

Washability (68%) appeared to be particularly relevant among the requirements placed on coatings by consumers (Q3, Table 2). For >50% of the respondents, it was also important that the coating is labeled and is not perceptible on the product.

## 4 Conclusions

The present study aimed to evaluate the potential of edible coatings for soft-skinned fruit with edible skins, by conducting a general survey and an exemplary application on cucumbers. The commercially available lipid-based edible coating used in this investigation, effectively build a tight barrier against gas diffusion, well-compared to that of commercial wax coatings. The application of this lipid-based coating on cucumbers drastically diminished the losses of fruit mass and the reduction of tissue stiffness. It also lowered CO_2_ release by about one third, attenuated respirational activity and, thus, prolonged shelf life. On the other hand, the application of lipid-based edible coating did not negatively affect fruit quality of the cucumber as indicated by the constancy of relevant quality indicators. The edible coating used certainly showed great potential to improve the quality maintenance of perishable horticultural products.

From the survey of consumer acceptance, it became obvious that many participants support the application of edible coatings as an alternative to plastic packaging. This highlights the relevance of developing suitable alternatives to plastic packaging for highly perishable fresh fruit and vegetables, prone to disproportionate food losses if offered completely unprotected. Important requirements for the successful use of edible coatings are washability, labelling and low perceptibility. In terms of washability, lipid-based coatings with relative low melting temperatures (40–60 °C) are highly suitable, because they can be easily removed with hot tap water.

## CRediT authorship contribution statement

**G. Rux:** Conceptualization, Methodology, Formal analysis, Investigation, Writing, Visualization, Supervision. **C. Labude:** Conceptualization, Methodology, Formal analysis, Investigation. **W.B. Herppich:** Writing, Writing – review & editing. **M. Geyer:** Conceptualization, Resources, Writing – review & editing, Project administration, Funding acquisition.

## Declaration of competing interest

The authors declare that they have no known competing financial interests or personal relationships that could have appeared to influence the work reported in this paper.

## Data Availability

Data will be made available on request.
